# Opioids for the management of dyspnea in cancer patients: a systematic review and meta-analysis

**DOI:** 10.1007/s10147-023-02362-6

**Published:** 2023-06-20

**Authors:** Yusuke Takagi, Junya Sato, Yoshihiro Yamamoto, Ryo Matsunuma, Hiroaki Watanabe, Masanori Mori, Takaaki Hasegawa, Yoshinobu Matsuda, Jun Kako, Yoko Kasahara, Sho Goya, Hiroyuki Kohara, Takeo Nakayama, Takashi Yamaguchi

**Affiliations:** 1 Department of Palliative Medicine, Teikyo University School of Medicine, 2-11-1 Kaga, Itabashi-ku, Tokyo, 173-8605 Japan; 2 Department of Pharmacy, International University of Health and Welfare Hospital, Nasu-Shiobara, Japan; 3 Department of Pharmacy, Komaki City Hospital, Komaki, Japan; 4 Department of Palliative Medicine, Konan Medical Center, Kobe, Japan; 5 Home Palliative Care Asunaro Clinic, Komaki, Japan; 6 Division of Palliative and Supportive Care, Seirei Mikatahara General Hospital, Hamamatsu, Japan; 7 Center for Psycho-Oncology and Palliative Care, Nagoya City University Hospital, Nagoya, Japan; 8 Department of Psychosomatic Internal Medicine, National Hospital Organization Kinki-Chuo Chest Medical Center, Sakai, Japan; 9 College of Nursing Art and Science, University of Hyogo, Kobe, Japan; 10 Department of Pharmacy, Hiroshima Prefectural Hospital, Hiroshima, Japan; 11 Department of Respiratory Medicine, Kinki Central Hospital of the Mutual Aid Association of Public School Teachers, Itami, Japan; 12 Department of Internal Medicine, Hatsukaichi Memorial Hospital, Hatsukaichi, Japan; 13 Department of Health Informatics, Kyoto University, Kyoto, Japan; 14 Department of Palliative Medicine, Kobe University Graduate School of Medicine, Kobe, Japan

**Keywords:** Opioid, Dyspnea, Cancer, Systematic review, Meta-analysis

## Abstract

Dyspnea is a prevalent symptom that significantly reduces quality of life of cancer patients. Palliative treatment is necessary when the symptoms do not respond to treatment for their cause. Opioids are widely used as pharmacological therapy, but evidence for individual agents is inconsistent. The purpose of this study was to evaluate the efficacy and safety of opioids for dyspnea in cancer patients. We searched the CENTRAL, MEDLINE, EMBASE, and ICHUSHI for studies using opioids for dyspnea in adult cancer patients reported by September 2019. Screening of the retrieved literature and assessment of risk of bias and outcomes were performed by two independent authors. A meta-analysis was performed on the primary endpoint, relief of dyspnea, and secondary endpoints including quality of life, somnolence as a side effect, and serious adverse events. Twelve randomized controlled trials were evaluated regarding relief of dyspnea. Somnolence and serious adverse events were evaluated in seven and four randomized controlled trials, respectively, but no randomized controlled trials were evaluable for quality of life. Overall, opioids were more effective than placebo for dyspnea (standardized mean difference − 0.43, 95% confidence interval [CI] − 0.75 to – 0.12). Although significant difference was found between systemic morphine and placebo in the drug-specific analysis, no significant difference could be detected in the other analyses. Systemic administration of opioids is more effective than placebo in relieving dyspnea in cancer patients. Robust evidence on the efficacy and safety of opioids on dyspnea in cancer patients is lacking, and further studies are needed.

**Supplementary Information:**

The online version contains supplementary material available at 10.1007/s10147-023-02362-6.

## Introduction

Dyspnea is a symptom that significantly reduces the quality of life of cancer patients [1], and 46–59% of cancer patients experience moderate to severe dyspnea [2]. Dyspnea is an independent adverse prognostic factor in cancer patients [3], and the frequency and severity of dyspnea increases in patients with deteriorating general condition and at the end of life [4, 5]. Shortness of breath on exertion is a physiological phenomenon that happens naturally in anyone, but dyspnea at rest or severe dyspnea on exertion restricts daily life and social functioning, deprives patients of independence, and causes frustration, anger, and depression [6]. Therefore, treatment for dyspnea is needed to maintain the patient's quality of life.

It is known that dyspnea in cancer patients is more likely to occur in patients with lung lesions (including metastatic tumors). To alleviate dyspnea in cancer patients, assessment of the causative pathology of dyspnea and treatment of the cause is a prerequisite. It is also important to consider adjustments in activities of daily living and the environment. When treatment for the cause is difficult or no more effective, pharmacological and non-pharmacological palliative therapies are used to alleviate symptoms [7, 8].

Opioids are widely used as pharmacological therapy to relieve dyspnea in cancer patients and are recommended as first-line pharmacological therapy by several guidelines [7, 9, 10]. Among them, the usefulness of systemic morphine for dyspnea in cancer patients has been widely reported, and in recent years, the usefulness of other opioids such as fentanyl and oxycodone has also been reported in some non-randomized studies [8]. However, currently available studies employ multiple rating scales, and their sample sizes are small and follow-up periods are relatively short. Therefore, guidelines and systematic reviews have not resulted in strong recommendations [7, 9, 10].

Recently, a number of studies in this area have been conducted and could improve clinical practice for dyspnea in cancer patients. In addition, existing systematic reviews have not adequately evaluated opioids by type [11, 12], and a detailed evaluation could provide insights to improve clinical practice for cancer patients with dyspnea. Therefore, we conducted this study to evaluate the efficacy and safety of each opioid for dyspnea in cancer patients.

## Patients and methods

### Registration and protocol

This systematic review and meta-analysis was registered with PROSPERO prior to the initial literature search according to the Preferred Reporting Items for Systematic Reviews and Meta-analysis (PRISMA) 2020 statement [13] (PROSPERO No. 201111127).

### Eligibility criteria and endpoints

We searched for studies of adult cancer patients treated with opioids for refractory dyspnea despite appropriate treatment for potentially reversible factors. Efficacy endpoints included relief of dyspnea as the primary endpoint, quality of life as a secondary endpoint, and somnolence and serious adverse events due to opioids as secondary safety endpoints. The degree of dyspnea was required to be measured by patients reported outcomes. Serious adverse events were defined as Common Terminology Criteria for Adverse Events (CTCAE) Grade 3 or higher or adverse events described as serious by the study authors. We included systemic administration of morphine, oxycodone, hydromorphone, and fentanyl, or morphine inhalation in the analysis. The study inclusion criteria for the primary outcome were as following: (1) If there were at least two randomized controlled trial (RCT)s, inclusion was completed. (2) If there were less than 2 RCTs, we included non-RCTs or observational studies with control groups. (3) If there were no RCTs/non-RCTs/observational studies with control groups, single-arm observational studies were considered for inclusion. Case reports or case series were excluded. Secondary outcomes were analyzed for studies that met the criteria for the primary outcome. At the time this meta-analysis was designed, there was insufficient evidence that the drugs used in the control arms (opioids and benzodiazepines) in the active control RCTs were literally "active," meaning more effective than placebo. Therefore, the primary analysis was conducted without distinguishing between placebo control and active control RCTs, and additional subgroup analyses were conducted to examine these differences. We selected literature in English and Japanese.

### Information source, search strategy

We searched the CENTRAL, MEDLINE, EMBASE, and ICHUSHI of the Japan Medical Abstract Society databases for literature reported up to September 23, 2019, using “opioid”, “dyspnea”, and “cancer” as the main keywords. Details of the search formula are shown in Supplementary Table 1. In addition, an up-date search using PubMed was conducted on November 15, 2020.

### Selection process

All abstracts and titles of the literature obtained from the search were independently screened as the primary screening by two authors (YT and YY for morphine, oxycodone, and fentanyl and RM and JS for hydromorphone and morphine inhalation). Full text was reviewed and assessed for eligibility in the secondary screening. In cases of disagreement regarding inclusion, a third author (HW for morphine, oxycodone, and fentanyl; MM for hydromorphone and morphine inhalation) was included to discuss and reach consensus, if necessary.

### Data collection process, data items

The two authors independently extracted data from the adopted literature. In case of disagreement, a consensus was reached through discussion including the third author, if necessary. Items examined covered the following: authors and year of publication, number of patients included, background disease, demographic profile of patients, method of allocation, method of blinding, drugs and administration route used in the intervention, outcome measurements, and timing of assessments.

### Study risk of bias assessment

Two independent authors assessed the included studies for risk of bias according to the Minds Manual for Guideline Development 2020 ver. 3.0 [14]. Selection bias (randomization, concealment), performance bias (blinding of subjects), detection bias (blinding of assessments), attrition bias (intention-to-treat analysis, incomplete outcome data), reporting bias (selective reporting of outcomes), early trial termination, and other biases were evaluated. Each was scored individually as low risk, moderate/unclear, or high risk. Disagreements were discussed with the third author and consensus was reached.

### Effect measures, synthesis methods

Continuous variables were combined for effect measures, and standardized mean differences were extracted with 95%CIs. When effects were evaluated at multiple timings, the result closest to the timing of the mode was taken as the representative value. Relative risks (RRs) were calculated for binary variables such as adverse events. The integrated analysis was basically based on a random-effects model, but for study groups with similar interventions, a sensitivity analysis with a fixed-effects model was also performed. Statistical software was Review Manager 5.4.

### Reporting bias assessment, certainty assessment

The risk of bias for the integrated data was assessed with reference to the risk of bias for each article. Statistical heterogeneity was quantified using the *I*^2^ statistic, which represents the proportion of total variation in the study due to heterogeneity rather than sampling error; heterogeneity was considered significant if *I*^2^ was greater than 50% and p less than 0.1. Other biases such as publication bias were also evaluated.

### Additional analyses

Because the effect of the drug of interest itself may not be adequately assessed in an integrated analysis in which placebo-controlled and active control studies were mixed, additional analyses were conducted for the placebo-controlled and active control study groups, respectively. Furthermore, we performed sensitivity analyses according to the way the outcome was expressed (absolute value vs. change from baseline) and the type of dyspnea (at rest or on exertion).

## Results

### Study selection

**Fig. 1:**
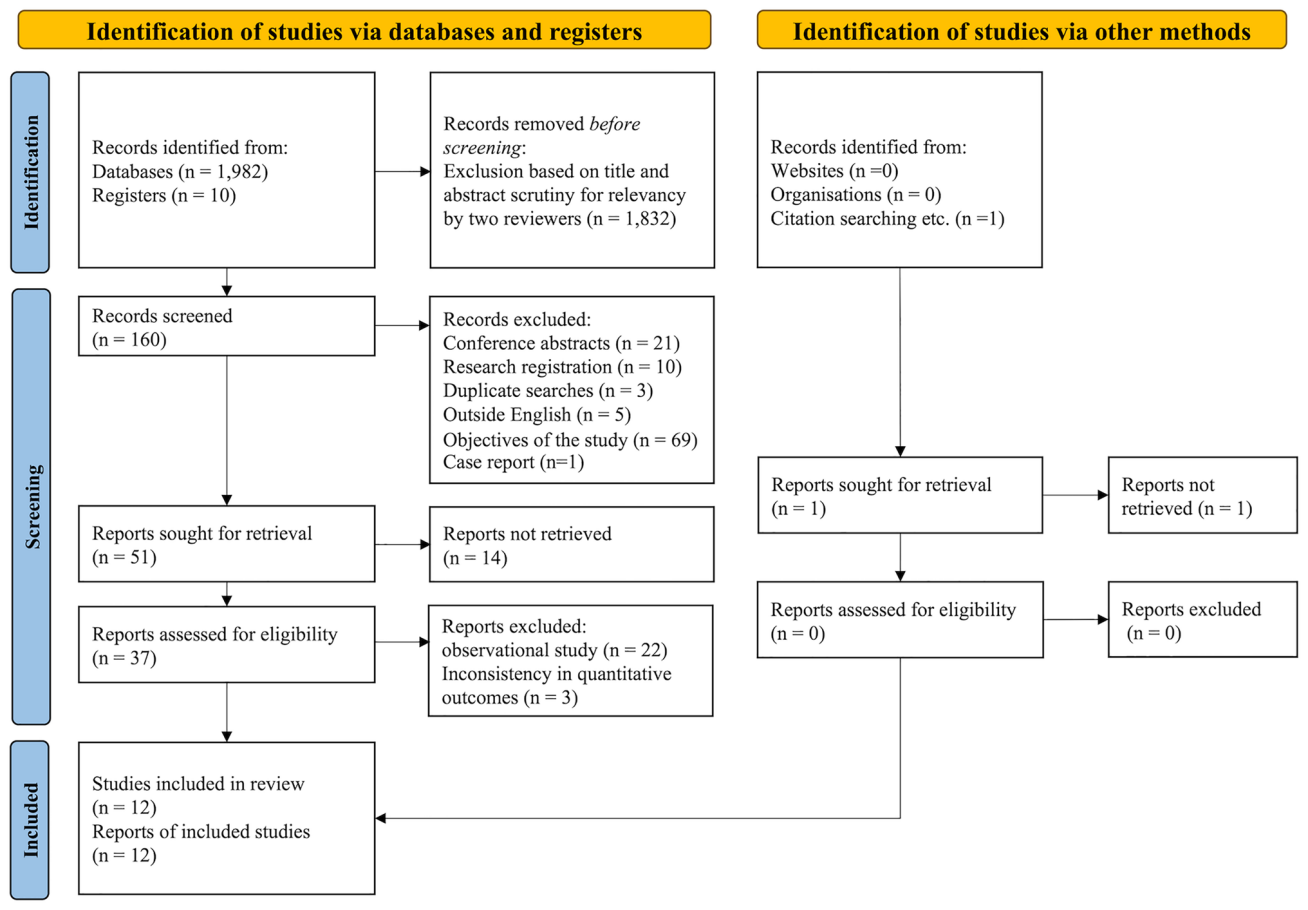
PRISMA 2020 flow diagram of the study

Twelve RCTs [15–26] evaluating relief of dyspnea were selected after a full-text review as a secondary screening (Fig. 1, Table 1). There was one RCT that evaluated quality of life [27], but it did not meet our eligibility criteria for RCTs because its control group employed non-pharmacologic interventions. We therefore handled this study as an observational study. There were seven RCTs [16–19, 21, 25, 26] that reported somnolence, and serious adverse events were reported in four RCTs [17–19, 23].

**Table 1:** List of selected randomized controlled trials

Author (pub date)	Interventions	Numver of patients	Dominant cancer type (N of patients)	Outcomes				Summary
				Dyspnea	QOL	Somnolence	SAE	
Bruera (1993) [15]	Mor (sc) vs. Placebo	10 (crossover)	Terminal cancer (lung 3)	●				VAS in Mor was significantly lower at 30, 45, and 60 min after the administration than Placebo
Mazzocato (1999) [16]	Mor (sc) vs. Placebo	9 (crossover)	Lung (7), breast (1), bladder (1)	●		●		VAS change from baseline at 45 min was significantly larger in Mor than Placebo
Bruera (2005) [17]	Mor (sc) vs. Mor (inhalation)	12 (crossover)	Lung (7), gastro-intestinal (2)	●		●	●	Found no significant difference in NRS at 60 min after administration between the two groups
Navigante (2006) [18]	Mor (sc) vs. midazolam (sc) vs. Mor + midazolam	101	Lung (30), breast (19), gynecological (14), sarcoma (12)	●		●	●	Showed no significant differences in modified Borg scale among subcutaneous morphine, midazolam and morphine plus midazolam groups at 24 and 48 h of administration
Charles (2008) [22]	HM (po/sc) vs. HM (inhalation) vs. Placebo	20 (crossover)	Lung (14), breast (2), renal (2), methotheli-oma (1), prostate (1)	●				Improvement from baseline in NRS 10 min after the end of intervention was not significantly different between the groups
Navigante (2010) [19]	Mor (po) vs. midazolam (po)	63	Lung (16), breast (15), head and neck (6)	●		●	●	NRS on the following day of administration was significantly better in the midazolam group
Hui (2014) [23]	Fen (sc) vs. Placebo	20	Breast (5), sarcoma (5), lung (4), urologic (3), gynecological (2)	●				Trend toward improvement from baseline walk in NRS at the walk after subcutaneous fentanyl, although no group comparison was made
Pinna (2015) [24]	Fen (tm) vs. Placebo	13 (crossover)	Lung (10), gastric (1), Breast (1), renal (1)	●			●	Showed no significant difference in the NRS between fentanyl and placebo at any time points
Simon (2016) [20]	Mor (po) vs. Fen (tm)	10 (crossover)	Lung (4), hematologic (2)	●				Found no significant difference in NRS change from baseline at 10 and 30 min post-dose between the two groups
Hui (2016) [25]	Fen (tm) vs. Placebo	20	Gastrointestinal (6), breast (5), lung (3)	●		●		Trend toward improvement from baseline in NRS 20 min after administration in the intranasal fentanyl group, although no between-group comparison was made
Hui (2017) [26]	Fen (tm) vs. Placebo	20	Lung (8), gastro- intestinal (3), urologic (3), breast (3)	●		●		NRS after a 6-min walk was not significantly different between the sublingual fentanyl and placebo
Yamaguchi (2018) [21]	Mor (po) vs. Oxy (po)	17	Lung (9), colorectal (2)	●		●		NRS change from baseline at 60 and 120 min post-dose was not significantly different between the two groups

*Fen* fentanyl, *HM* hydromorphone, *Mor* Morphine, *NRS* numerical rating scale, *Oxy* oxycodone, *po* oral administration, *QOL* quality of life, *SAE* severe adverse events, *sc* subcutaneous injection, *tm* transmucosal administration, *VAS* visual analog scale

### Study characteristics

A total of 326 patients were evaluated from 12 RCTs, with a median number of patients in each study of 18.5 (minimum 9, maximum 101). Approximately 40% of the patients had lung tumors (primary lung cancer or lung metastases). The route of systemic administration of opioids was oral or subcutaneous injection, and transmucosal for fentanyl studies. Placebo control was employed in seven studies, while active control was used in the others. Comparisons between opioids were three, and comparisons with benzodiazepines were performed in two studies. The timing of the efficacy assessment was 30–60 min after the drug administration in most studies. Four (80%) of the five studies using fentanyl evaluated outcomes before and after the 6-min walk test.

Overall, lung cancer was relatively common among the patients included, with some patients having concomitant pleural effusions and lymphangitis carcinomatosa. Some studies also reported patients with COPD, interstitial lung disease, heart failure, and bronchial asthma, but these non-malignant complications were generally limited in a minority of patients up to about 20%, except in Navigante's study [19] (57% with interstitial lung disease and 37% with pulmonary micro-embolism).

### Risk of bias in studies

**Fig. 2:**
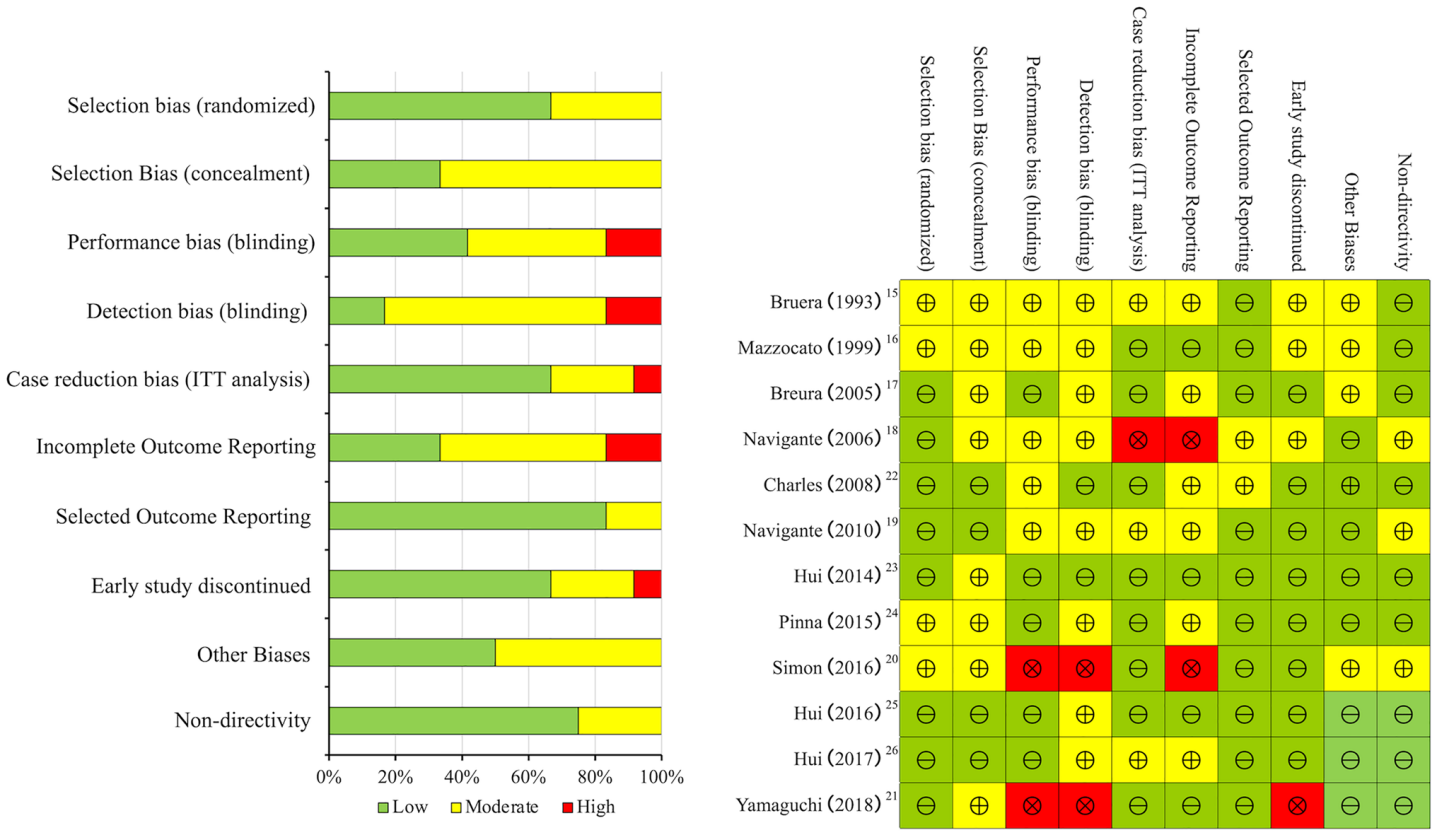
Bias risks of selected randomized controlled studies. ITT, intention-to-treat. The left side shows the distribution of the studies by bias, and the right side details the risk of bias for each individual study

Three studies were assessed to have a low risk of bias [23, 25, 26], all of which were studies of systemic fentanyl administration with 6-min walk tests by the same investigator. Six studies were classified to have a moderate and three to have a high risk of bias [18, 20, 21] (Fig. 2).

### Primary outcome: relief of dyspnea

Seven RCTs evaluated relief of dyspnea with morphine [15–21], one with oxycodone [21], one with hydromorphone [22], five with fentanyl [20, 23–26], and one with morphine inhalation [17] (with overlap). Of these, studies in which the degree of dyspnea was assessed as a continuous variable and for which a CI could be estimated were included in the integrated analysis. Four trials [15, 16, 20, 21] with systemic morphine, one trial [21] with oxycodone, one trial [22] with hydromorphone, and five trials [20, 23–26] with fentanyl met these criteria. Trials comparing opioids with other opioids [20, 21] were excluded from the integrated analysis of opioids overall.

**Fig. 3:**
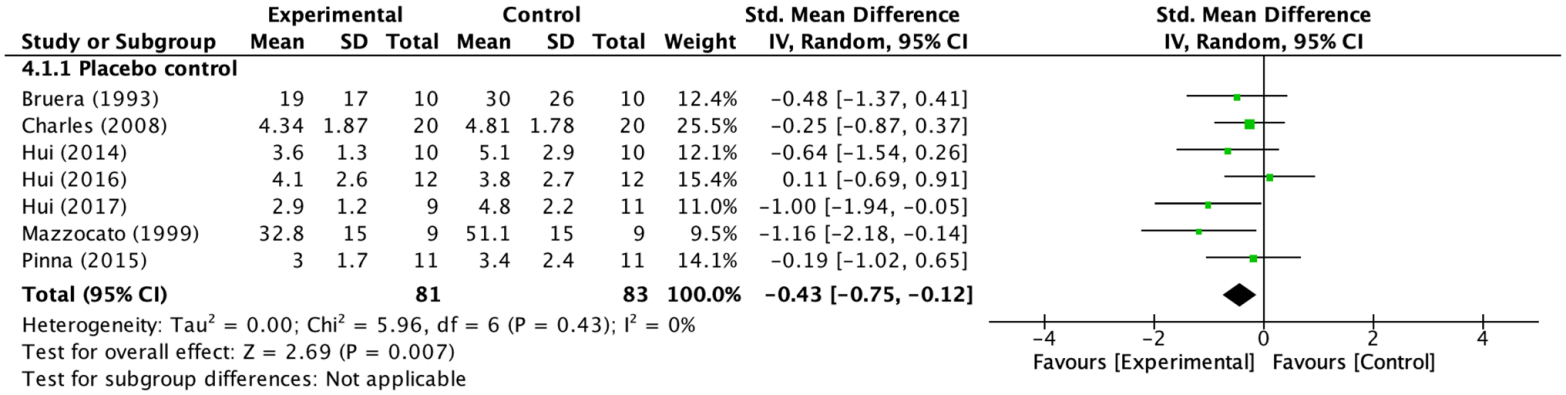
Forest plots for palliation of dyspnea. *CI* confidence interval,* IV* inverse variance, *SD* standard deviation, *Std* standard. Mean and SD represent the dyspnea measures; Total represents the number of patients; Experimental and Control represent the opioid intervention and placebo, respectively

The integrated analysis of opioids included seven trials, all of which were placebo-controlled. The effect on dyspnea was significant with a standardized mean difference of − 0.43 (95% CI − 0.75 to  −  0.12) (Fig. 3).

**Fig. 4:**
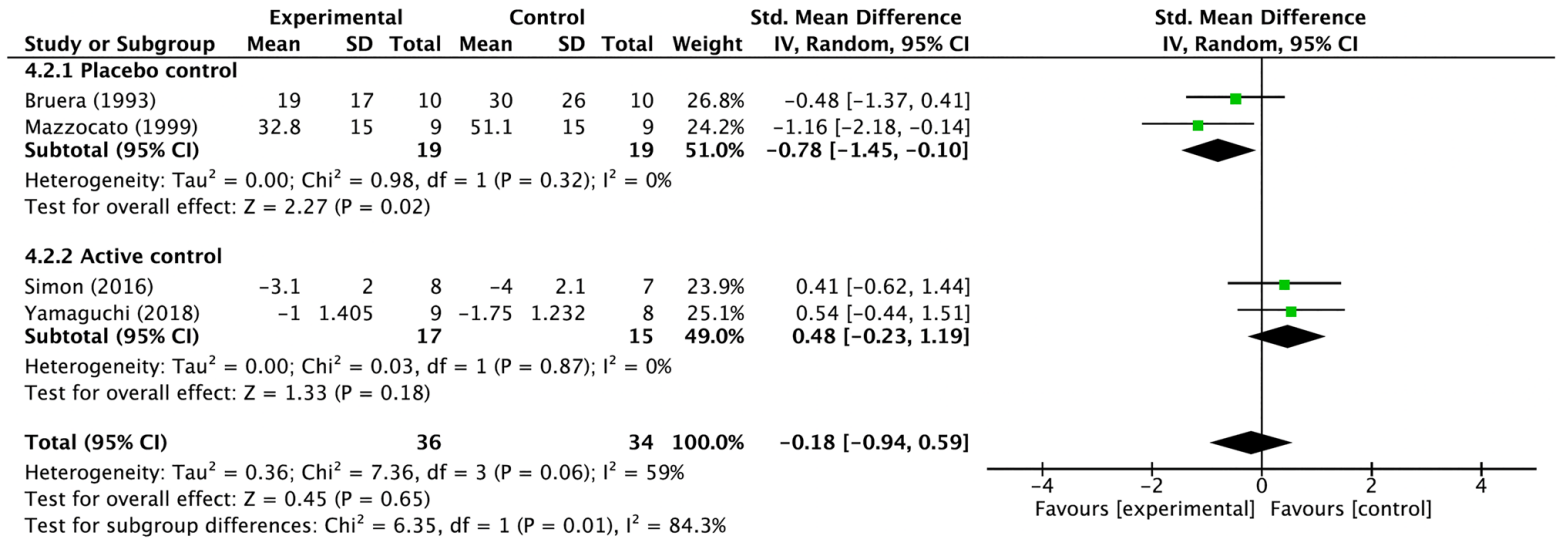
Forest plots for palliation of dyspnea for the morphine subgroup. *CI* confidence interval; *IV* inverse variance, *SD* standard deviation, *Std* standard. Mean and SD represent the dyspnea measures; Total represents the number of patients; Experimental corresponds to morphine and Control represents placebo or active control

When the four studies of systemic morphine administration were combined, the effect of morphine on dyspnea compared to placebo or other interventions was not significant with a standardized mean difference of − 0.18 (95% CI − 0.94 to 0.59) (Fig. 4). When the two placebo-controlled trials [15, 16] were combined, the standardized mean difference was significant at − 0.78 (95% CI − 1.45 to − 0.10). For the two active control trials [20, 21], the standardized mean difference was 0.48 (95% CI − 0.23 to 1.19). The difference in effect between the two subgroups was significant (*I*^2^ = 84.3%, *p* = 0.01).

**Fig. 5:**
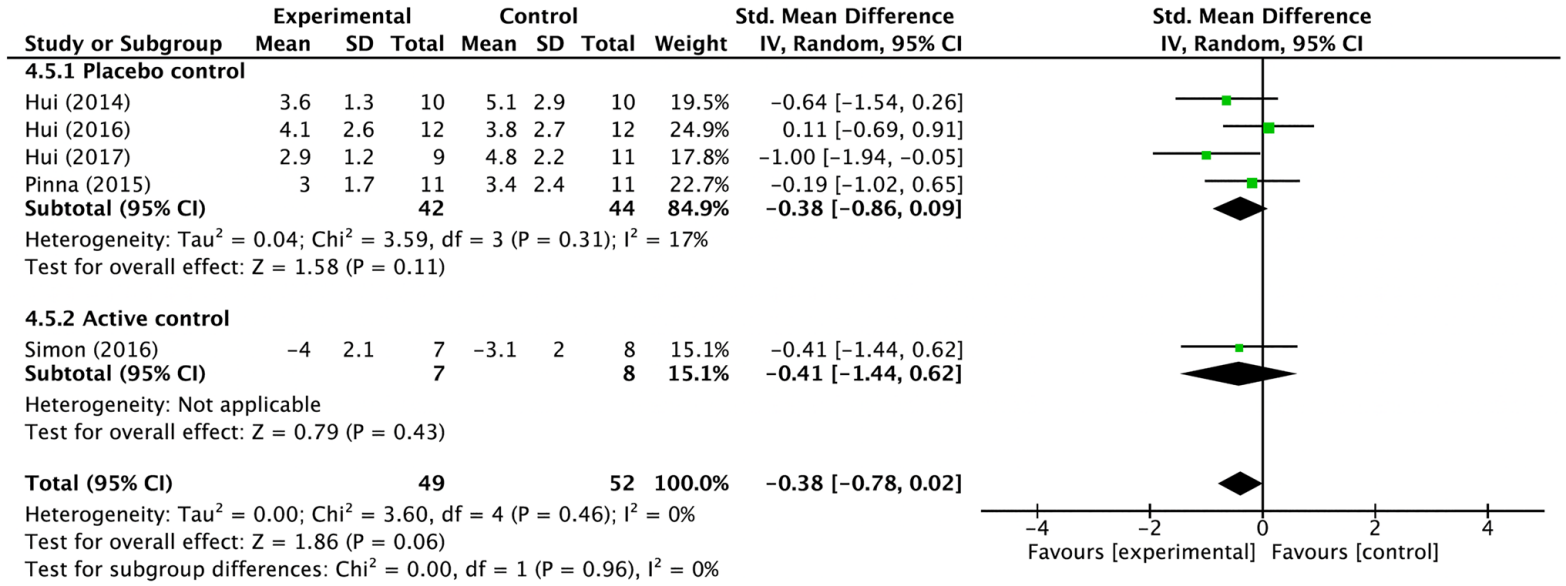
Forest plots for palliation of dyspnea for the fentanyl subgroup. *CI* confidence interval, *IV* inverse variance, *SD* standard deviation, *Std* standard. Mean and SD represent the dyspnea measures; Total represents the number of patients; Experimental corresponds to fentanyl and Control represents placebo or active control

The combined results of the five studies examining the effect of fentanyl on dyspnea showed a standardized mean difference of − 0.38 (95% CI − 0.78 to 0.02) compared to the other interventions, which was not significant (Fig. 5). The standardized mean difference remained insignificant at − 0.38 (95% CI − 0.86 to 0.09) when four placebo-controlled trials [23–26] were combined, and the difference in effects between the two subgroups was not significant (*I*^2^ = 0, *p* = 0.96).

The heterogeneity among the seven trials in the integrated analysis of opioids overall was low (*I*^2^ = 0%, *p* = 0.43), and no significant publication bias was found in the funnel plot (Supplementary Fig. 1). Extracting the four trials that evaluated the efficacy of systemic morphine, we found modest heterogeneity between the placebo-controlled trials and the actual drug-controlled trials. Although the heterogeneity of the five trials that examined the efficacy of fentanyl was low, the majority of trials evaluated dyspnea associated with 6-min walks, considered a high degree of non-directness for problems with dyspnea in real-world cancer care.

### Secondary outcomes

#### Quality of life

One RCT evaluated the improvement in quality of life with opioids administered for dyspnea [27], but it was a three-arm comparison of systemic morphine, acupuncture, and a combination of morphine and acupuncture, and did not meet the eligibility criteria for this meta-analysis. The European Organization for Research and Treatment of Cancer Quality of Life Questionnaire Core 30 (EORTC QLQ-C30) score for the morphine systemic arm of the study did not change significantly before or after morphine administration.

### Somnolence

There were five RCTs evaluating somnolence with morphine [16–19, 21], one with oxycodone [21], zero with hydromorphone, two with fentanyl [25, 26], and one with morphine inhalation [17]. There were no nonrandomized studies for hydromorphone or morphine inhalation. Of these, studies with identified number of patients with somnolence were included in the integrated analysis. Four trials [16, 18, 19, 21] with systemic morphine, one trial [21] with oxycodone, and two trials [25, 26] with fentanyl met this criterion.

**Fig. 6:**
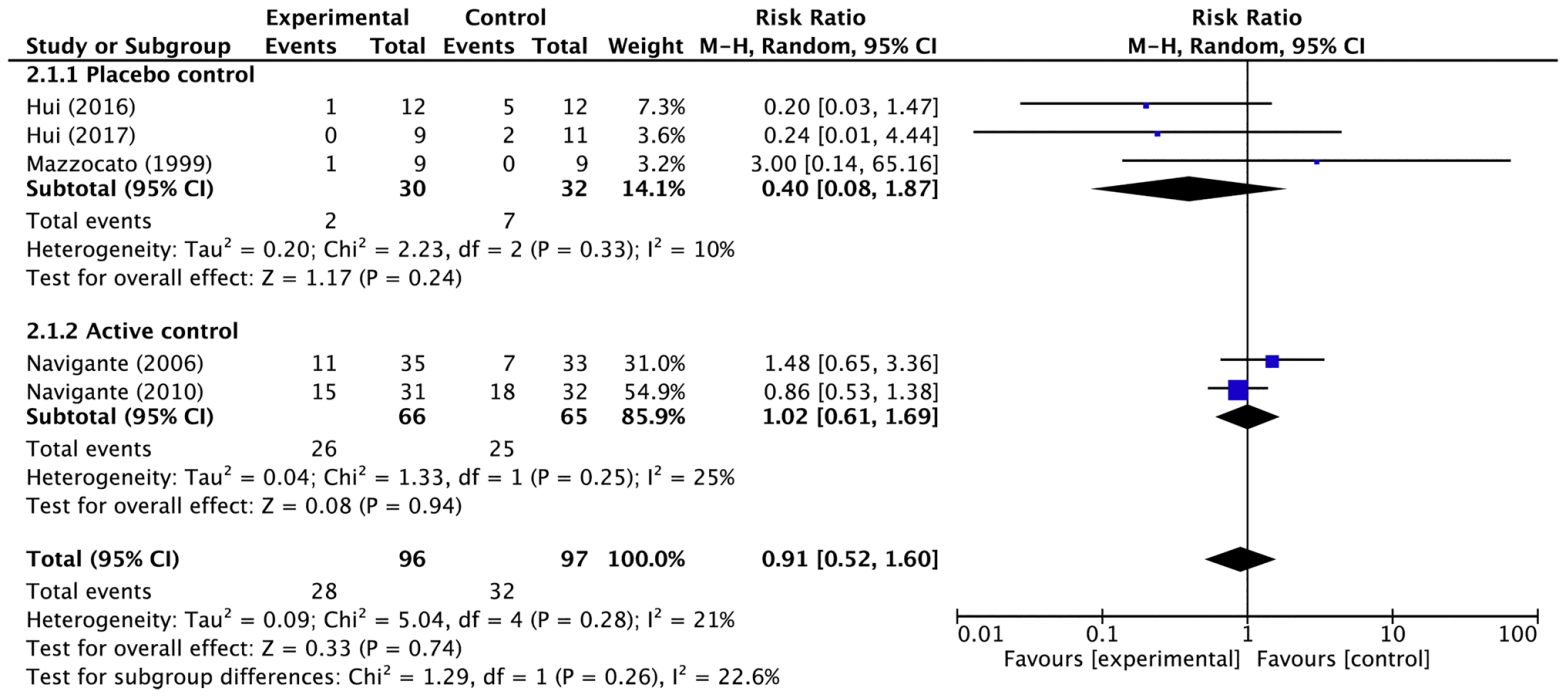
Forest plots for somnolence. *CI* confidence interval, *M-H* Mantel–Haenszel. Events and Total represents the number of patients; Experimental corresponds to opioids and Control represents placebo or active control

**Fig. 7:**
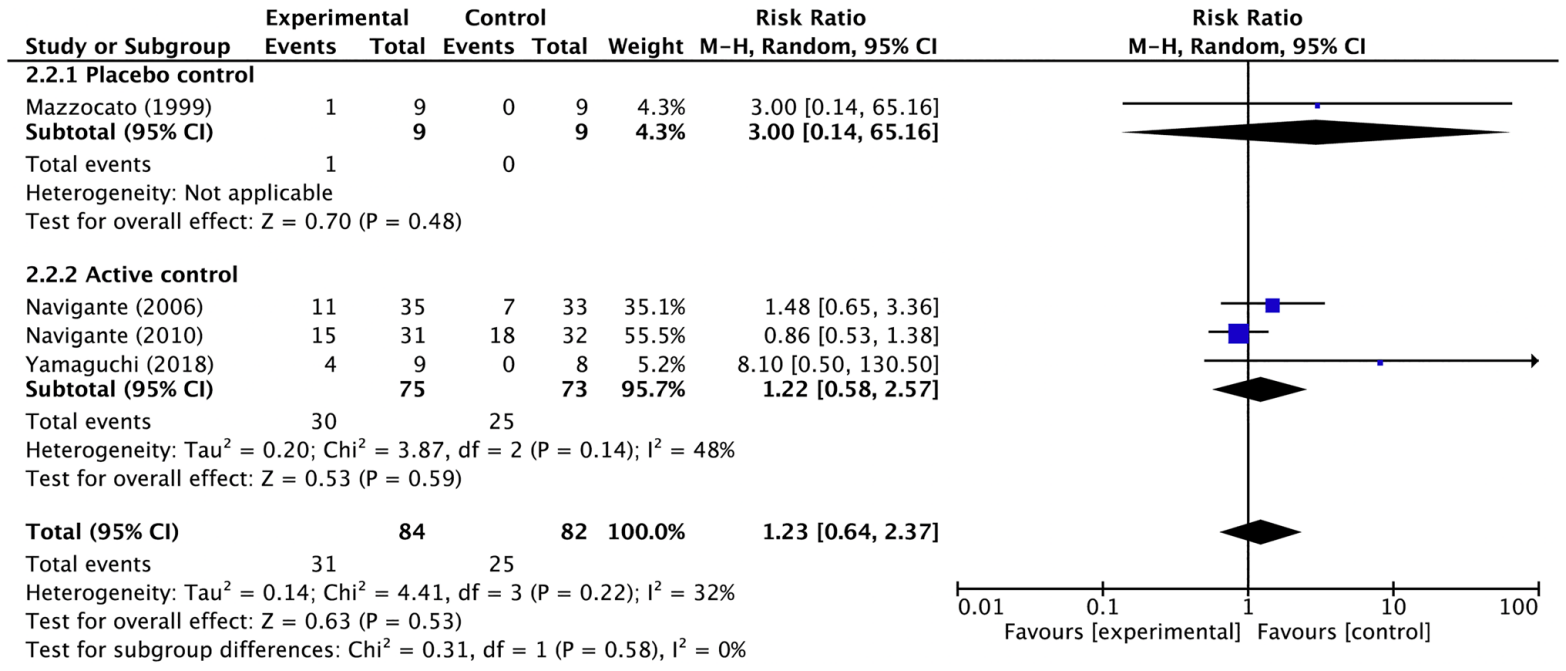
Forest plots for somnolence for the morphine subgroup. *CI* confidence interval, *M-H* Mantel–Haenszel. Events and Total represents the number of patients; Experimental corresponds to morphine and Control represents placebo or active control

**Fig. 8:**
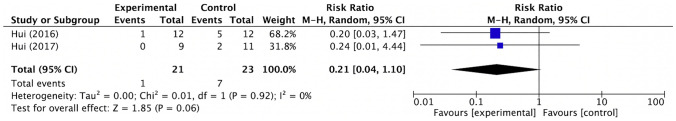
Forest plots for somnolence for the fentanyl subgroup. *CI* confidence interval, *M-H* Mantel–Haenszel. Events and Total represents the number of patients; Experimental corresponds to fentanyl and Control represents placebo

In an integrated analysis of five trials, excluding a RCT comparing opioids among themselves [21], there was no significant increase in opioid-induced somnolence compared with placebo or other agents (Fig. 6). Subgroup analyses such as systemic morphine versus other interventions (risk ratio: 1.23; 95% CI 0.64–2.37) (Fig. 7), systemic morphine versus placebo or active control (Fig. 7), and fentanyl versus other interventions (risk ratio: 0.21; 95% CI 0.04–1.10) did not show significant difference (Fig. 8).

The heterogeneity among the five trials in the integrated analysis of opioids overall was low (*I*^2^ = 21%, *p* = 0.28), and no significant publication bias was found in the funnel plot (Supplementary Fig. 2). Extracting the four studies that evaluated the efficacy of systemic morphine, we found modest heterogeneity in the active-control studies, possibly due to the effect of Yamaguchi’s study [21], in which a particularly high proportion of somnolent patients were observed in the morphine group. The two fentanyl trials included in the integrated analysis had low potential for inconsistency.

### Severe adverse events

Among RCTs evaluating serious adverse events due to opioids administered for dyspnea, 3 [17–19] evaluated morphine, 0 oxycodone, 0 hydromorphone, 1 fentanyl [24], and 1 morphine inhalation [17]. Trials with a control group in which the number of serious adverse events was specified were included in the synthesis analysis. Two trials for systemic morphine [18, 19] and one for fentanyl [24] met this criterion. No trials were included in the integrated analysis for oxycodone, hydromorphone, or morphine inhalation.

**Fig. 9:**
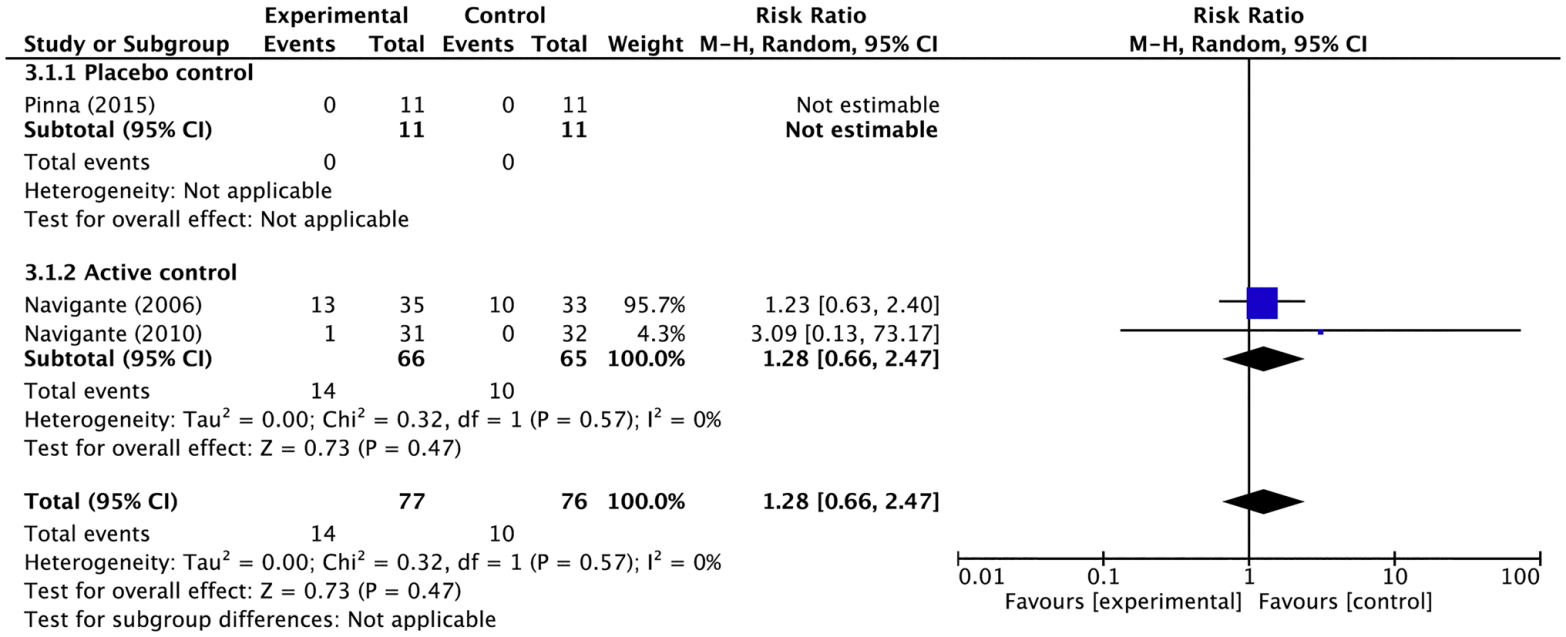
Forest plot for severe adverse events. *CI* confidence interval, *M-H* Mantel–Haenszel. Events and Total represents the number of patients; Experimental corresponds to opioids and Control represents placebo or active control

In the integrated analysis of the above three trials, there was no significant increase in serious adverse events with opioids compared to placebo or other drugs (Fig. 9). The combined analysis of the two trials examining serious adverse events with systemic morphine showed a risk ratio of 1.28 (95% CI 0.66–2.47) compared to other interventions, which was not significant.

Heterogeneity among the three trials in the integrated analysis of opioids overall was low (*I*^2^ = 0%, *p* = 0.57), and there were not enough studies to allow an assessment regarding publication bias. The two trials included in the integrated analysis for systemic morphine administration were less inconsistent.

### Additional analyses

In the main analysis, the post-intervention value of dyspnea intensity was used as the outcome measure, and the results were consistent in a sensitivity analysis in which that was replaced by the change from baseline (Supplementary Fig. 3). Sensitivity analyses of studies that assessed improvement in dyspnea at multiple time points showed a consistent trend in results (Data not shown). Because many of the RCTs with fentanyl also addressed dyspnea on exertion, a sensitivity analysis was performed for trials that assessed improvement in dyspnea on exertion [23–26], but the results were not different (Supplementary Fig. 4). Sensitivity analyses with fixed-effects models were also performed for study groups with similar interventions, but no change in trend or significance of results was found (Data not shown).

## Discussion

Twelve RCTs with more than 300 patients were included in this integrated analysis. The number of trials, especially for systemic morphine and fentanyl, was large compared to other opioids. However, the individual trials were mainly small, with around 10–20 cases per arm, and more than half of the trials included in this study had a risk of bias related to blinding. Dyspnea is a strong poor prognostic factor in cancer patients, making them extremely vulnerable and prone to irreversible deterioration. Given the high barriers to conducting a large double-blind trial in this context, the current evidence seems to meet the acceptable level of evidence needed to conduct a meta-analysis.

Regarding efficacy on dyspnea, an integrated analysis of opioids as a whole showed superiority of opioid over placebo. In examining individual agents, morphine was shown to be effective in comparison with placebo. Despite some risk of bias, the two studies provide evidence for the clinical questions of interest, especially in that they target dyspnea at rest. The efficacy of fentanyl was marginal, and oxycodone and hydromorphone could not be adequately studied due to the small number of cases.

While placebo-controlled studies supported the efficacy of morphine, our result showed no clear difference between morphine and active control. While this result leaves open the possibility that other agents might also have effectiveness for dyspnea, it is likely to be essentially influenced by the small sample size. In particular, there was not enough evidence for oxycodone and hydromorphone to conclude their efficacy against dyspnea. For fentanyl, despite placebo-controlled studies conducted in a larger number of cases than for morphine, the results of the integrated analysis slightly failed to reach the level of significance. Most studies examining the effects of fentanyl have focused on exertion-related dyspnea, and this difference in target setting may have contributed to the results of the current analysis. Dyspnea at rest and exertional dyspnea in cancer patients may differ in mechanism, and the latter can be caused by relatively minor impairments such as decreased lung diffusion capacity or muscle weakness, even if they do not have significant respiratory failure that leads to dyspnea at rest. Most studies examining fentanyl have applied a temporary load to patients with relatively higher respiratory function by walking for 6 min, which may result in less benefit than in other studies. Although a sensitivity analysis comparing the effects of opioids on resting dyspnea and exertion-related dyspnea showed no clear difference, further evidence is needed to clarify which patients are more effective with opioids in real-world clinical practice. It should be noted that the results of this analysis do not immediately prohibit the use of opioids other than morphine for dyspnea, but the rationale is much weaker than that for morphine.

There was no clear evidence that opioids significantly increase somnolence or severe adverse events. Typical side effects of opioids are known to include somnolence, nausea and vomiting, constipation, and delirium. Although dyspnea is a strong poor prognostic factor in cancer patients and early death has been observed in many studies, there is no evidence that opioid use increases early death, and it appears feasible to use opioids in poor prognosis patients with caution about the general risks associated with their use. On the other hand, the use of opioids (rescue or regular administration) and patient backgrounds in the included studies were diverse, so the utmost caution should be paid to the patient's physical condition and the dose of opioids that may increase the adverse events.

As in previous reported meta-analyses, there was not enough evidence to draw definitive conclusions for each outcome other than improvement in dyspnea. In particular, very few studies included improvement in quality of life as an outcome, making it impossible to conduct a clinically meaningful analysis. Although some studies examined patient preferences [24], there was no robust evidence that such measures were surrogates for quality of life, and it was unclear whether they represented the overall balance of benefits and side effects that we were aiming for, so the authors discussed and decided not to include them in this analysis. Conducting RCTs in palliative care field is often more difficult than in other fields due to various issues such as patient vulnerability, irreversibility, obtaining consent, and psychological issues [28]. In addition to developing new methods for conducting RCTs, there is a need to accumulate reliable evidence for alternatives to RCTs, such as large prospective cohort studies that are appropriately adjusted for confounding.

Limitations other than sample size and the heterogeneity of the studies include the lack of inquiries to the authors of each study regarding the details of conducting the trial and individual patient data, which are necessary for a detailed assessment of the risk of bias. Although these were due to practical limitations, some studies were excluded because they reported only a median NRS for dyspnea [17], and obtaining further data from the authors could have yielded more reliable analyses than were obtained here.

In conclusion, although evidence for the use of opioids to improve dyspnea remains insufficient, systemic administration of opioids appears to be more effective than placebo in relieving dyspnea in cancer patients. Further accumulation of evidence for various conditions manifesting dyspnea is needed to provide direct evidence of patient benefits, including quality of life.

## Supplementary Information

Below is the link to the electronic supplementary material.

Supplementary file1 (DOCX 756 KB)
