# Supplementary material for: Opioids for the management of dyspnea in cancer patients: a systematic review and meta-analysis
Source: Int J Clin Oncol. 2023 Jun 20;28(8):999–1010. doi: 10.1007/s10147-023-02362-6 (PMC10390357; doi:10.1007/s10147-023-02362-6)

Supplementary Table 1. Detailed search formula.

| [(opioid* or opiate* or algopan or avinza or biopon or buprenorphine or cofapon or codeine or cyclobenzaprine or fentanyl or heroin or hydrocodone or hydromorphone or kadian or laudanon or laudanum or laudopan or levorphanol or loperamide or meperidine or methadone or morphine or nepenthe or omnopon or opial or opium or opana or opon or oposal or oxycodone or oxycontin or oxymorphonmme or pantopon* or papaveretum or pavon or pentazocine or percocet or pethidine or propoxyphene or tapentadol or tetrapon or tramadol or vicodin)] AND [(dyspnea* or dyspnoea* or dyspneic or short* of breath or breathless*) OR ((difficult* or laboured or labored) adj (respiration or breathing))] AND [(cancer* or tumor* or tumour* or neoplas* or carcinoma* or adenocarcinoma* or malignan* or oncolog* or sarcoma*)] AND [(aerosol* or atomiser* or inhalation or inhalator* or inhaler* or intravenous or nebulise* or oral or parenteral or po or subcutaneous or spray* or vaporiser*)] |
| --- |

Supplementary figure 1. Funnel plot for palliation of dyspnea.

SE, standard error; SMD, standard mean difference.

Supplementary figure 2. Funnel plot for somnolence.

SE, standard error; SMD, standard mean difference.

Supplementary figure 3. Sensitivity analysis for studies with before-and-after comparison.

CI, confidence interval; IV, inverse variance; SD, standard deviation; Std., standard. Mean and SD represent the dyspnea measures; Total represents the number of patients; Experimental and Control represent the opioid intervention and placebo, respectively.

Supplementary figure 4. Sensitivity analysis for dyspnea at rest or exertional dyspnea.

CI, confidence interval; IV, inverse variance; SD, standard deviation; Std., standard. Mean and SD represent the dyspnea measures; Total represents the number of patients; Experimental and Control represent the opioid intervention and placebo, respectively.


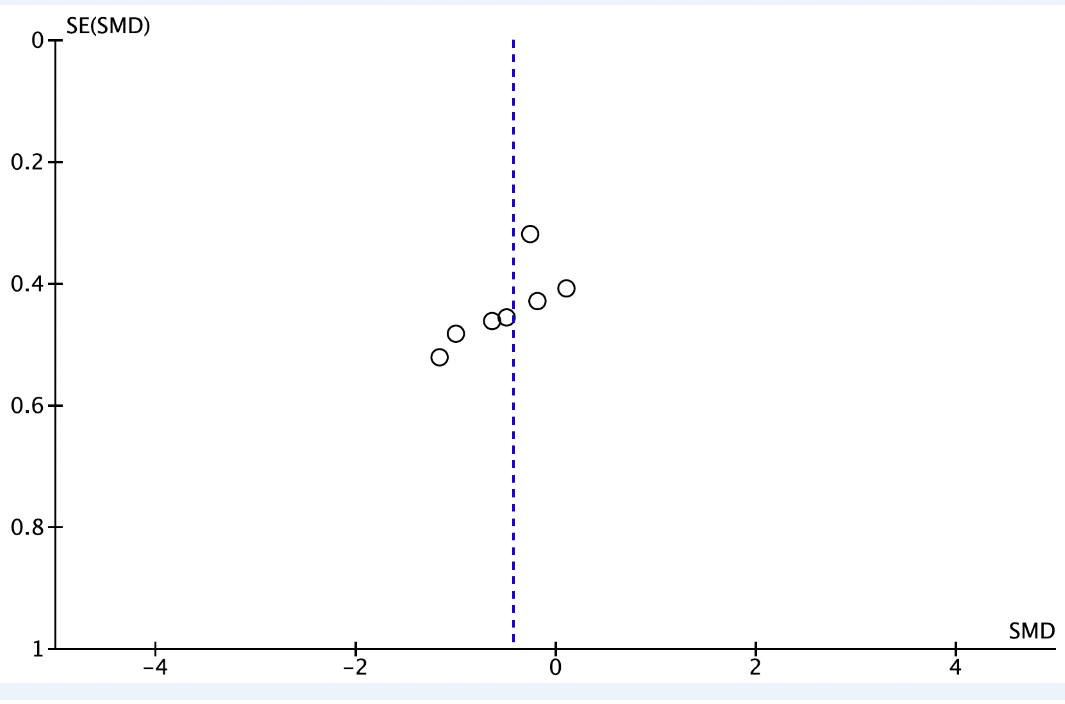


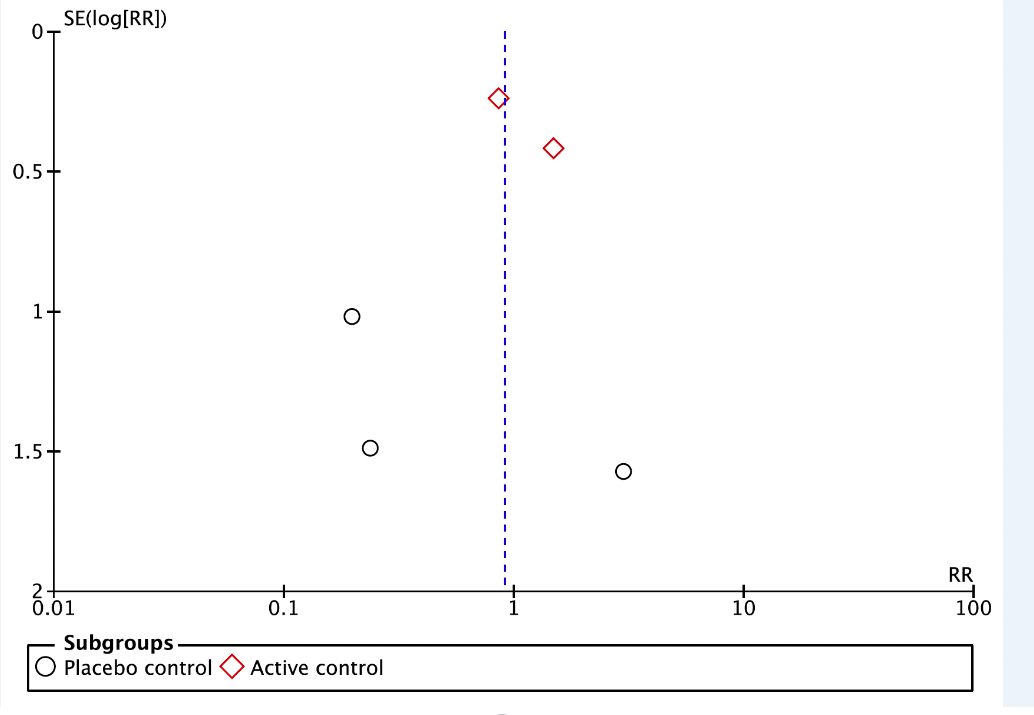


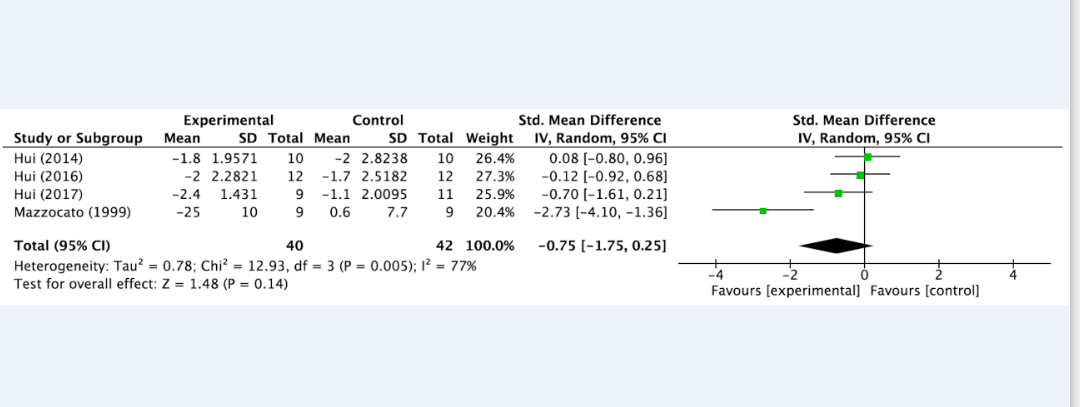


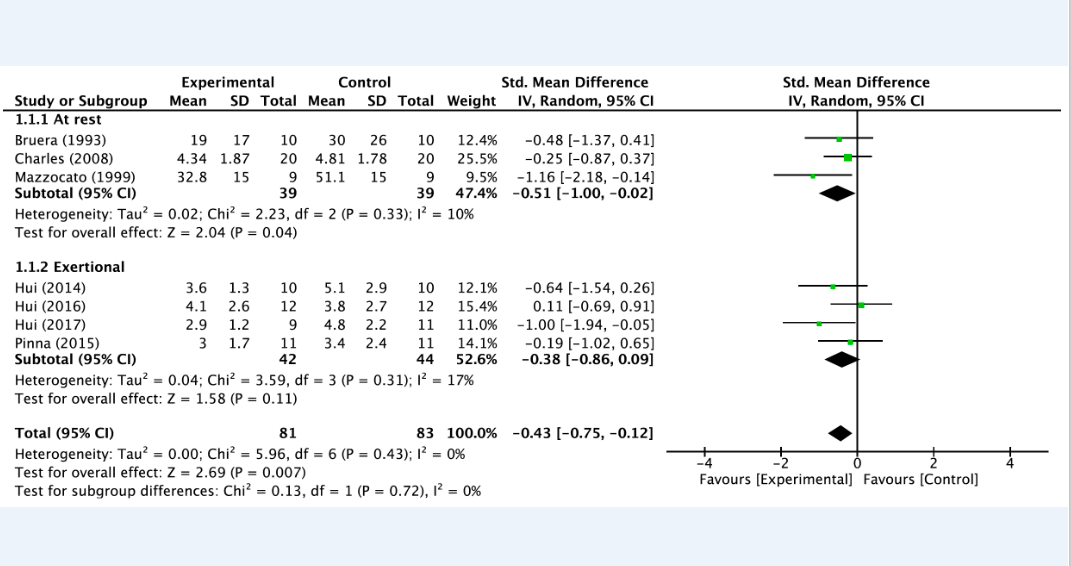

Supplement: Supplementary file 1 — Supplementary file1 (DOCX 756 KB) [file 10147_2023_2362_MOESM1_ESM.docx]
